# News headline generation based on improved decoder from transformer

**DOI:** 10.1038/s41598-022-15817-z

**Published:** 2022-07-08

**Authors:** Zhengpeng Li, Jiansheng Wu, Jiawei Miao, Xinmiao Yu

**Affiliations:** University of Science and Technology Liaoning, Anshan, China

**Keywords:** Computational science, Computer science, Information technology, Scientific data, Software

## Abstract

Most of the news headline generation models that use the sequence-to-sequence model or recurrent network have two shortcomings: the lack of parallel ability of the model and easily repeated generation of words. It is difficult to select the important words in news and reproduce these expressions, resulting in the headline that inaccurately summarizes the news. In this work, we propose a TD-NHG model, which stands for news headline generation based on an improved decoder from the transformer. The TD-NHG uses masked multi-head self-attention to learn the feature information of different representation subspaces of news texts and uses decoding selection strategy of top-k, top-p, and punishment mechanisms (*repetition-penalty*) in the decoding stage. We conducted a comparative experiment on the LCSTS dataset and CSTS dataset. Rouge-1, Rouge-2, and Rouge-L on the LCSTS dataset and CSTS dataset are 31.28/38.73, 12.68/24.97, and 28.31/37.47, respectively. The experimental results demonstrate that the proposed method can improve the accuracy and diversity of news headlines.

## Introduction

News headline generation (NHG)^1–5^ has been an important task in natural language processing (NLP), in recent years. NHG model can be divided into two categories: extractive and abstractive. The extractive directly selects several important words from the news text and rearranges them to form a news headline^6^. The abstractive uses advanced natural language processing algorithms to generate news headlines using techniques such as paraphrasing, synonymous substitutions, and sentence contractions. Since the neural network method has been applied to news headline generation, the neural network-based abstractive news headline generation model^7–10^ has recently shown great performance.

In recent years, encoder-decoder-based neural network models have been widely used in text summarization, mechanical fault detection^11^, etc. It is worth emphasizing that, the abstractive neural network model based on encoder-decoder^12–16^ has been proved to have a good performance on LCSTS dataset^17^, DUC-2004 dataset, and other data sets. The model based on a transformer effectively solves the problem of insufficient parallel ability of sequence-to-sequence models. The abstractive headline generation method can produce words that are not found in the original text, but this method may also make the generated news headlines out of the original facts^18^. As the recurrent neural network has the sequence coding characteristic that the information previously input will be gradually forgotten as time goes by, the intermediate semantics lack some significant information^19^, which leads to the headlines generated in the decoding process deviating from the main idea of the news text. Moreover, abstractive methods do not specifically process nonimportant or sub important text; that is, some nonimportant semantic information will be preserved with the same importance as feature semantic information when generating headlines, and there is noise interference.

**Figure 1:**
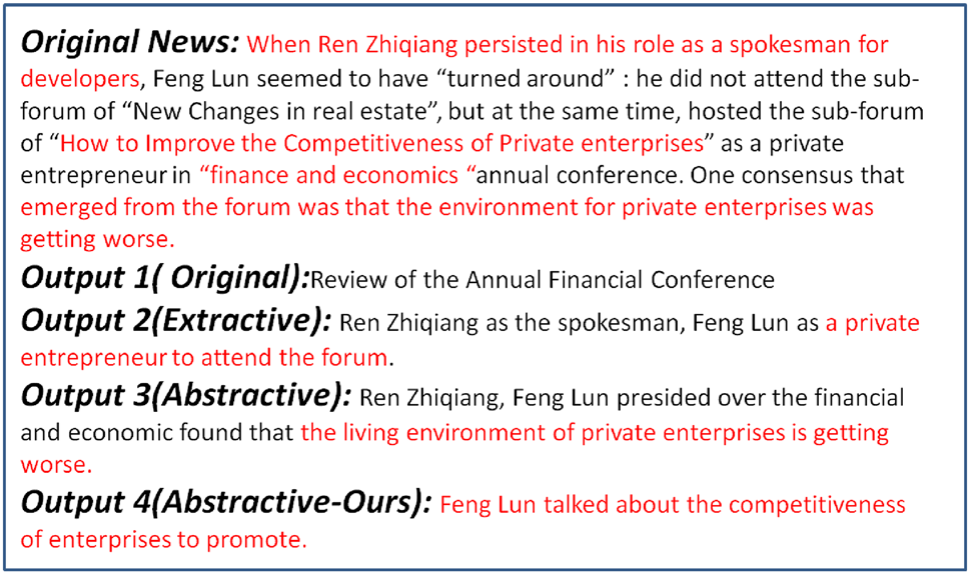
Comparison of output different models on a news document. Original New is the original news content. Output 1 (Original) is the news headline in the dataset. Output 2 (Extractive) is generated by the TextRank model. Output 3 (Abstractive) is generated by the sequence-to-sequence model. Output 4 (Abstractive-Ours) is generated by the TD-NHG model.

For example, as shown in Fig. 1, when the news is condensed, the extractive news headline generation directly extracts some semantic information. It can be observed that the TD-NHG model filters the semantic information in the original news, abandon the explicit information of “When Ren Zhiqiang persisted in his role as a reporter for developers” in the original news, and selects salient semantic information which is more important to the context semantic information, as the output of the generation. Outputs 3 and 4 choose two salient semantic pieces of semantic information as the output of the headline generation: “the living environment of private enterprises is getting worse” and “the competitiveness of enterprises to promote”, respectively. Compared with the original news text, it is found that the forum not only refers to the poor living environment of private enterprises but also how to improve their competitiveness in the case of large environmental changes.

Our contributions can be summarized as follows: (1) This paper proposes the TD-NHG model to solve the NHG problem. (2) We introduce masked multi-head self-attention into news headline generation and design a decoding selection strategy that integrates top-k, top-p, and punishment mechanisms to select important semantic information and generate news headlines. (3) We evaluate our proposed TD-NHG model on the LCSTS dataset and CSTS dataset. The experimental results show that the TD-NHG model is similar to or even exceeds the baseline model when dealing with NHG tasks.

## Background and related work

In the early 1980s, natural language generation gradually became a hot research field. In the 1980s and 1990s, a statistical language model was proposed to generate news headlines by analyzing word frequency, text location information, and text length, although this model is easy to implement, it cannot learn the complete semantic information in paragraphs. In 2004, Mihalcea et al. proposed TextRank^20^, which is a sort method based on a graph model. In this method, news text is divided into several words, and a TextRank network graph is constructed by taking these words as nodes and the number of co-occurrences within a certain range between words in news text as edges. The PageRank algorithm is used to update the graph until convergence, and the news headlines are composed of words with high ranking.

The sequence-to-sequence model is an end-to-end neural network. The sequence-to-sequence model is composed of five parts: document, tokenizer, encoder, attention, and decoder. The tokenizer segments the document into a series of words. The encoder is used to encode the word vector sequence into the hidden state of each word, and the weights of each word are calculated by attention. The decoder calculates the probability of each word in the vocabulary as an output word and uses a search algorithm to obtain news headlines. The sequence-to-sequence abstractive model often ignores the secondary important semantic information in feature semantic information extraction, and the parallel ability of the model is poor. Zhou et al.^21^ proposed the text summary generation method based on an improved sequence-based sequence model, which is composed of an input layer, hidden layer, and output layer and introduces a copy mechanism to solve the problem of out-of-vocabulary (OOV)^14^ words in the process of summary generation, but there is still room for further improvement of accuracy.

With T5^22^, STEP^23^, BART^24^ and other large-scale multitask pre-training models proposed, each NLP task has reached a new SOTA. Recurrent neural networks and sequence-to-sequence models have gradually been replaced by models based on the transformer. For example, BERT is a bidirectional pre-training model. BERT achieves SOTA in a variety of more than 10 NLP tasks by using a large number of unlabeled text training language models through unsupervised methods. This large-scale pre-training model has reached an amazing number of parameters. The BERT of 12 layers has approximately 110 M parameters, which can be trained on a single GPU. BERT of 24 layers and even more layers has more than 340 M parameters, which can only be run on TPU. This large-scale pre-training model has reached an amazing number of parameters. The BERT of 12 layers have approximately 110 M parameters, which can be trained on a single GPU. BERT with 24 layers and even more layers have more than 340 M parameters, which can only be run on TPU, and the scale of the computation is too large for the average researcher to handle. Ordinary researchers cannot afford to consume large amounts of computing power. The TD-NHG model used a decoding selection strategy integrating top-k, top-p, and punishment mechanisms (*repetition-penalty*) in the decoder stage, which achieved a good effect on News headline generation. In addition, it runs perfectly in parallel on a single GPU.

## Model

### Problem definition and overview

News headline generation (NHG) aims to train a neural network model to map a text into a short text headline^25^. The input of the NHG model is $X = \left\{ {x_{1} ,x_{2} ,\ldots,x_{i} } \right\}$, and the output news headline is $Y = \left\{ {y_{1} ,y_{2} ,\ldots,y_{j} } \right\}$. The vocabulary used by the NHG model is $V = \{ V_{1} ,V_{2} ,\ldots,V_{i} \}$. In this paper, the generation probability of TD-NHG can be formulated as

1 $$P(Y|X;\theta ) = \prod\limits_{j = 1}^{N} {P(y_{{\text{j}}} |X,y_{{1:{\text{j - 1}}}} ;\theta )}$$

where $\theta$ is the model parameter of the news headline generation.

**Figure 2:**
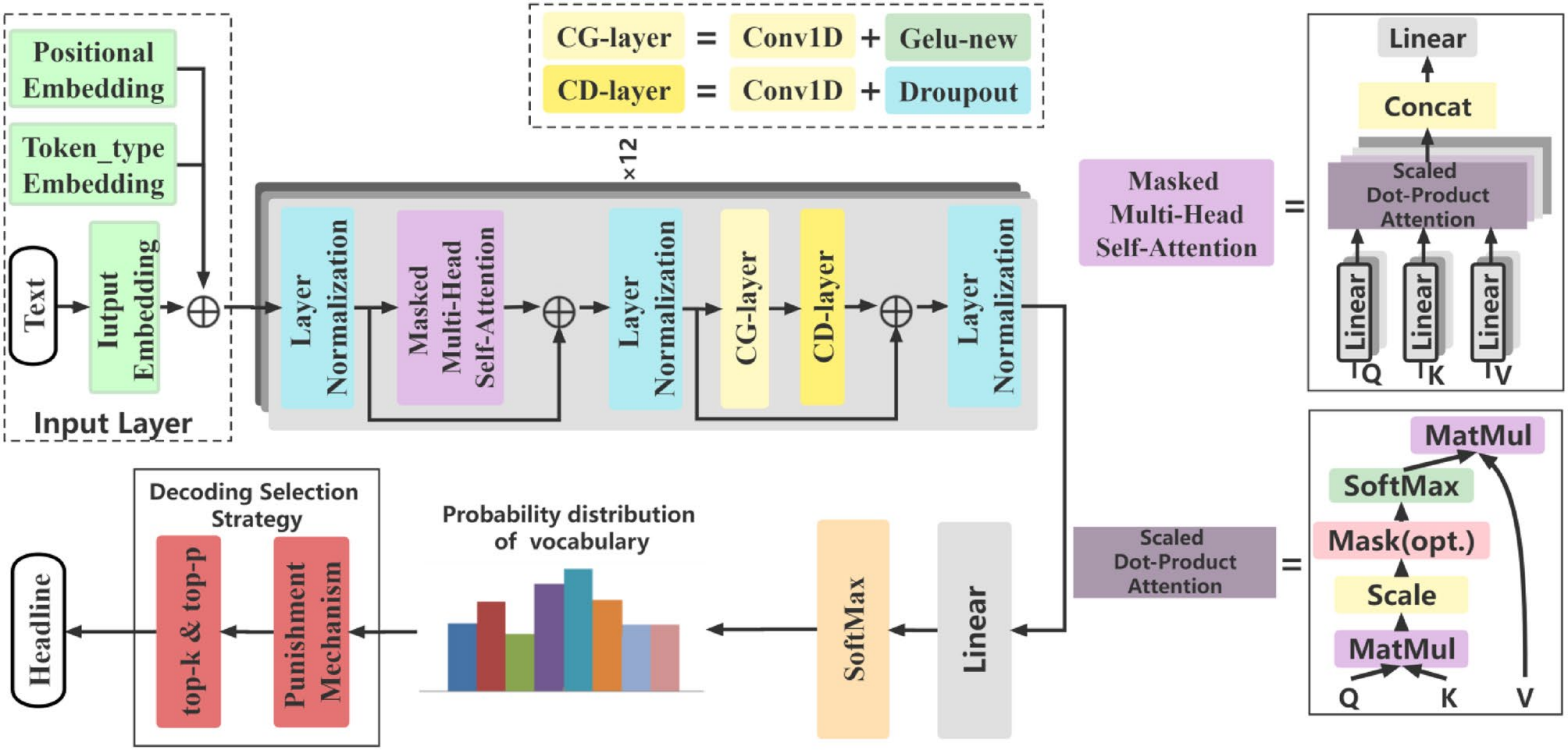
TD-NHG model diagram.

The transformer model is composed of an encoder and decoder, and both are stacked by parts called the “transformer module”, for example, encoding module, decoding module, attention module, normalization module, etc. Much of the subsequent work attempts to remove encoders or decoders, in other words, researchers stack multiple transformer modules and train or pre-training them using large text and considerable computing power. TD-NHG model is an autoregressive model with 12 transformer-decoder layers. The TD-NHG model is divided into three main parts: the input module of the news headline generation, generation module based on improved transformer-decoder, decoding selection strategy, and punishment mechanism. The model is shown in Fig. 2.

### Input module of news headline generation

The input module of the NHG model includes three parts. The first part is document representation (*D*), which is composed of [CLS], news text, [SEP], news headlines, and [SEP]. *D* is embedded into d-dimensional vector space by an embedding layer to obtain *E(D)*, such as formula (2),

2 $$E\left( {Input} \right) = \left\{ {E(D)} \right\},$$

The second part is the qualifier (*Z*). In addition to the five commonly used qualifiers [Space], [UNK], [CLS], [SEP], and [MASK], the TD-NHG model introduces [Content] and [Title] to effectively distinguish the news headline and news content. Input embedding layer embeds *Z* into embedding matrix.

The third part is positional embedding and segment embedding. The transformer model does not mark the order of the input words. To solve a positional information problem, the TD-NHG model adds positional embedding (*PE*) and segment embedding (*SE*) in the input layer, in which the dimension of *PE* and *SE* are consistent with that of input embedding (*IE*). The *PE* vector determines the relative distance between different tokens in a sentence. The formula is as follows (3) and (4),

3 $$PE\left( {pos_{n} ,2 \times ind} \right) = \sin \left( {{{pos_{n} } \mathord{\left/ {\vphantom {{pos_{n} } {\left( {10000^{{\frac{2 \times ind}{{d_{\bmod el} }}}} } \right)}}} \right. \kern-\nulldelimiterspace} {\left( {10000^{{\frac{2 \times ind}{{d_{\bmod el} }}}} } \right)}}} \right),$$

4 $$PE\left( {pos_{n} ,2 \times ind + 1} \right) = \cos \left( {{{pos_{n} } \mathord{\left/ {\vphantom {{pos_{n} } {\left( {10000^{{\frac{2 \times ind}{{d_{\bmod el} }}}} } \right)}}} \right. \kern-\nulldelimiterspace} {\left( {10000^{{\frac{2 \times ind}{{d_{\bmod el} }}}} } \right)}}} \right),$$

where $pos_{n} \in {\mathbb{R}}^{Len \times d}$ refers to the absolute position of the *n*-th word in the original sentence, *Len* is the maximum length of position information, and *d* represents the dimension of position embedding, *ind* is the dimension. TD-NHG model uses sine encoding when dealing with words in even positions, and sine encoding when dealing with words in odd positions. $E_{PE}$ is the location embedding matrix, and size is the maximum sequence length multiplied by embedding dimension, which is initialized with normal distribution to improve the readability of the headline. The output of the model input module is defined as formula (5),

5 $$E\left( {Output} \right) = E(D + Z) + E_{PE} \, (D + Z) + E_{SE} (D + Z),$$

$E\left( {Output} \right) \in {\mathbb{R}}^{{B \times L \times d_{e} }}$, where $B$ is the batch-size of the training model. *L* set to 512, which is the length of the input sequence, and $d_{e}$ is the dimension of the embedding representation. $E(D + Z)$ represents the token embedding of news ( including news content and headlines) and qualifier of the input model, $E_{PE} \, (D + Z)$ represents the position embedding of news and qualifier of the input model, $E_{SE} (D + Z)$ represents the segment embedding of news and qualifier of the input model.

### Generation module based on improved transformer-decoder

Input the output of the input module (*E(Output)*) into the generation module based on the improved transformer-decoder, which is normalized by layer normalization (*LN*). The normalized word vector is transmitted to masked multi-head self-attention. The attention layer mainly alleviates the complexity of the neural network. The attention layer does not need to input all *E(Output)* into the neural network for calculation. On the contrary, attention selects some task-related information to input into the neural network, which is similar to the idea of a gating mechanism in the RNN model. The attention mechanism is essentially an addressing process. By giving a task-related query vector (*Q*), calculating the attention distribution of *Q* and Key (*K*), and attaching it to Value (*V*), then the attention value is obtained.

The scaled dot-product attention used in the generation module based on improved transformer-decoder is optimized by adding scale and mask operations based on attention. The self-attention mechanism is the different attention of a single sequence at different positions, which is used to calculate the representation of the sequence. In this paper, *d*_*e*_ is used to represent the output dimension of the self-attention mechanism, and $\{ W^{Q} ,W^{K} ,W^{V} \} \in {\mathbb{R}}^{m \times d}$ is used to represent the trainable parameter matrix. Then, the context representation can be obtained according to the following calculation process,

6 $$Attention(Q,K,V) = SoftMax\left( {\frac{{(QK^{T} )}}{{\sqrt {(d_{k} )} }}} \right)V,$$

An additional scaling factor $\sqrt {d_{k} }$ is introduced, and the difference between additive attention and dot-product attention is minuscule when the value of $\sqrt {d_{k} }$ is small. However, if $\sqrt {d_{k} }$ increases, the dot-product value is large; as a result, the gradient after softmax^26^ is tiny, which is not conducive to backpropagation, so scaling is performed on the result.

**Figure 3:**
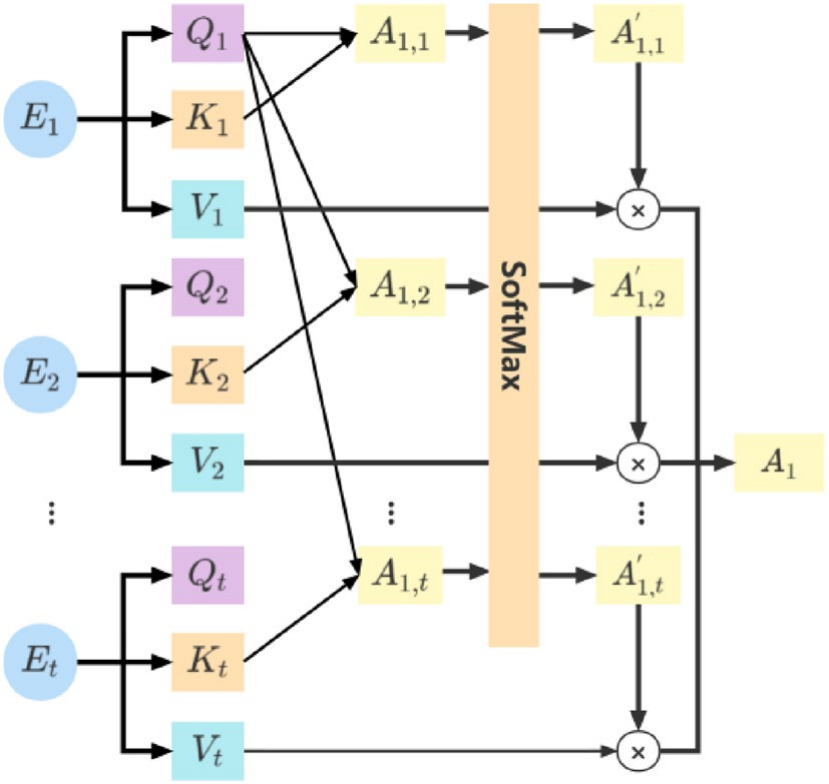
*A*_*1*_ context vector calculation method.

As shown in Fig. 3, $E = \left[ {E_{1} ,E_{2} ,E_{3} , \ldots ,E_{i} , \ldots ,E_{n} } \right]$ denotes the characteristic representation vector of the *i*-th time. When given the query vectors $Q = \left[ {Q_{1} ,Q_{2} ,Q_{3} , \ldots ,Q_{i} , \ldots ,Q_{t} } \right]$ and $K = \left[ {K_{1} ,K_{2} ,K_{3} , \ldots ,K_{i} , \ldots ,K_{t} } \right]$, the similarity $A_{1,i}$ of $Q_{i}$ and $K_{i}$ is calculated, $A_{1,i}$ calculation method as formula (7),

7 $$A_{1,i} = \frac{{Q_{1} K_{i} }}{\sqrt d },$$

The new weight $A_{1,i}^{^{\prime}}$ is calculated by softmax, the context vector $A_{i}$ is obtained, $A_{i}$ represents the context vector at the *i*-th time,

8 $$A_{i} = \sum\limits_{i = 1}^{n} {A_{1,i}^{^{\prime}} V_{i} } .$$

Multi-Head Self-Attention provides multiple representation subspaces for Attention. In each attention, with different *Q*, *K*, and *V* weighting matrices, each matrix is generated by random initialization. Then, the word embedding is projected into different representation subspaces by training.

The news headline generation task generates words in the news headline in turn, that is, the *i*-th word is generated before the (*i*+*1*)-th word is generated. The Masked operation prevents the *i*-th word from knowing the information after the (*i*+*1*)-th word. *Q*, *K*, *V* matrices are calculated by input matrix. Then calculate the product of *Q* and *K* and multiply the product of *Q* and *K* with matrix *V* to get the output. For the model to learn more subspace information, TD-NHG model uses mask multi-head self-attention to deal with different parts of the feature representation separately. Then, the self-attention result of the *i-th* subspace is shown in formula (9),

9 $$Space_{i} = Attention\left( {QW^{{\left( {Q_{i} } \right)}} ,KW^{{\left( {K_{i} } \right)}} ,VW^{{\left( {V_{i} } \right)}} } \right).$$

The self-attention results of each head are spliced, and the matrix $W^{dr}$ is used for multi-space fusion. Finally, the final result of mask multi-head self-attention is obtained, as shown in formula (10),

10 $$MultiHead = Concat\left( {Space_{1} ,Space_{2} , \ldots ,Space_{i} } \right)W^{dr} .$$

Finally, the final context representation matrix $M \in {\mathbb{R}}^{t \times d}$ is obtained by layer normalization. The multi-head self-attention mechanism assigns different weights to the feature vectors at different time steps. Therefore, in conclusion, the large weights are allocated to a few key feature vectors, while most irrelevant feature vectors can only obtain a small amount of weight. This method effectively solves the problem of the equal contribution of feature vectors of each time step and captures the long distance dependence and time dynamic correlation of feature vectors of each time step.

### Decoding selection strategy and punishment mechanism

Beam search will abandon some unimportant semantic information in the search process, greatly reducing space consumption and improving time efficiency, but beam search does not pay enough attention to sub important semantic information and may produce repetitive, meaningless text that makes headlines inaccurate. TD-NHG model proposes a new decoding selection strategy, which utilizes $top{\text{-}} k$ sampling and the $top{\text{-}} p$ method to introduce the temperature parameter (*t*) in the softmax calculation process to change the vocabulary probability distribution, making it more biased toward high probability words.

11 $$P(x\left| {x_{1:i - 1} } \right.) = \frac{{exp(u_{t} /t)}}{{\sum_{t\prime } exp(u_{t\prime } /t))}},$$

where *u* represent logits, and $t \in [0,1)$.

Currently, the entered sentence has a fixed size hidden state, TD-NHG model will generate the hidden state of the *t* word based on the hidden state of the input sentence and the first to *t-*1 words ($x_{1:i - 1}$) generated previously. Finally, the vocabulary probability distribution ($P(x\left| {x_{1:i - 1} } \right.)$) of the *t* word was obtained by softmax function.

In the process of model decoding, select the *k* tokens with the highest probability from $P(x\left| {x_{1:i - 1} } \right.)$ distribution and sum their probabilities to get $\sum {P(x\left| {x_{1:i - 1} } \right.)}$, where $x \in V^{k}$. Vocabulary probability distribution of the *t* word is updated to $p^{\prime}(x\left| {x_{1:i - 1} } \right.)$,

12 $$p^{\prime}(x\left| {x_{1:i - 1} } \right.) = \frac{{p(x\left| {x_{1:i - 1} } \right.)}}{{\sum {P(x\left| {x_{1:i - 1} } \right.)} }},$$

**Figure 4:**
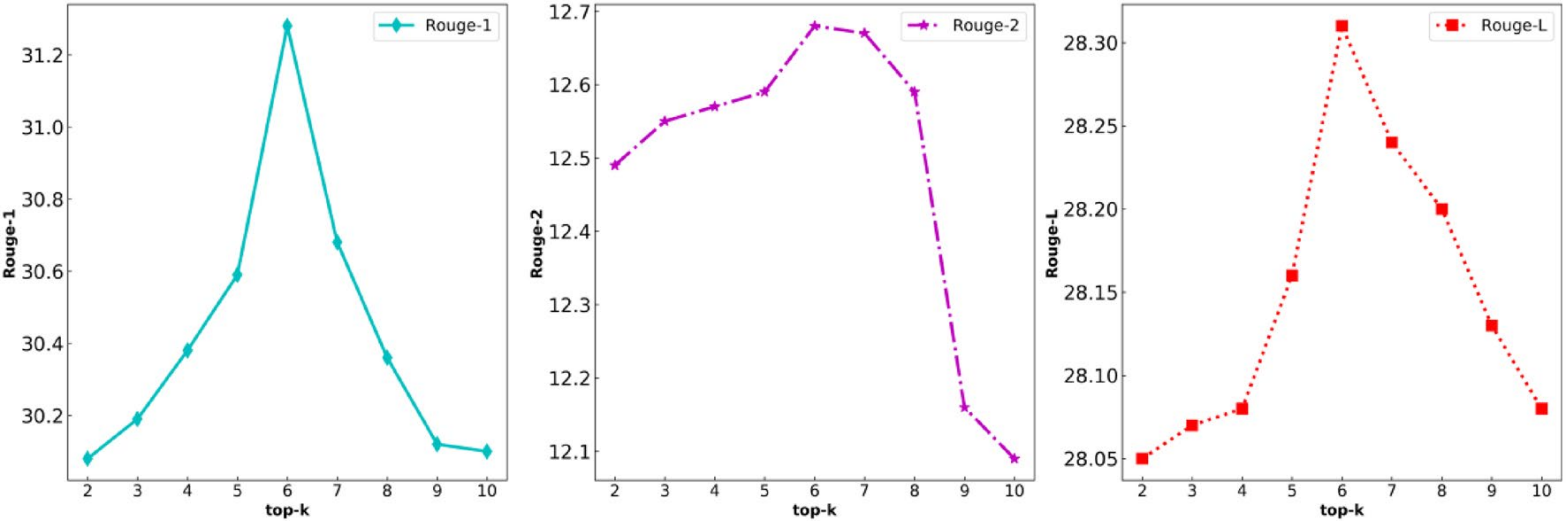
The influence curve of top-k on Rouge ($top{\text{-}} p = 0.3$).

**Figure 5:**
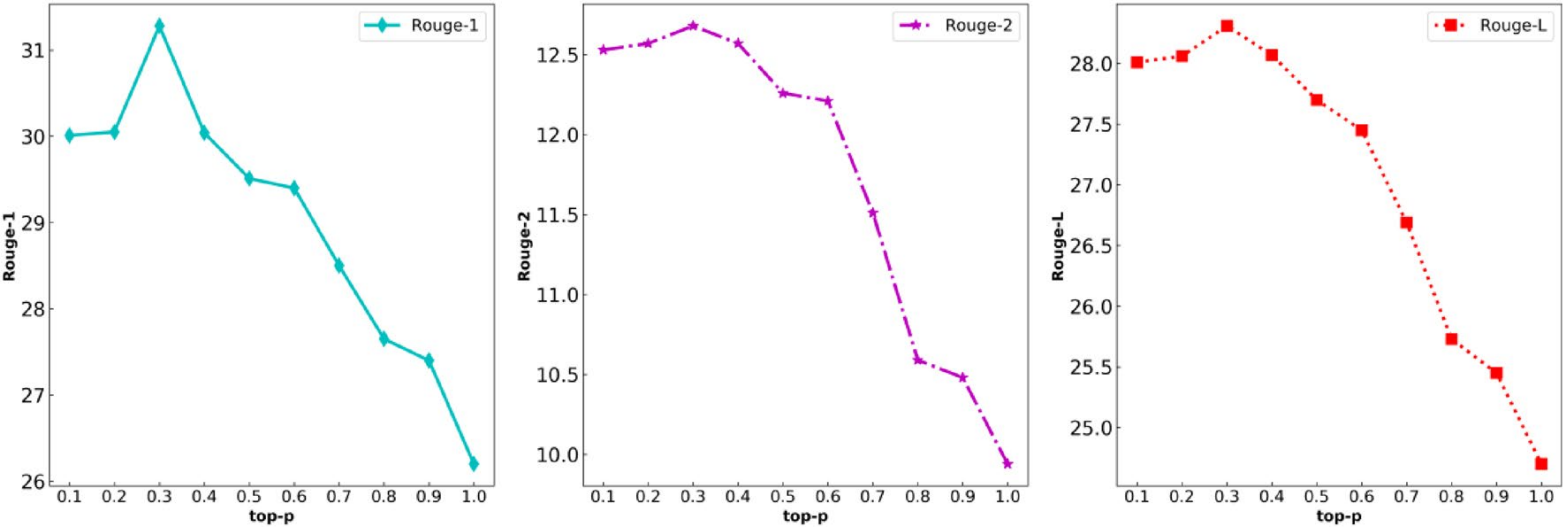
The influence curve of top-p on Rouge ($top{\text{-}}k = 6$).

Finally sample a token from the candidate set ($p^{\prime}(x\left| {x_{1:i - 1} } \right.)$) as an output token. However, the problem with top-k sampling is that constant *k* is a given value in advance. For sentences with different lengths and contexts, the model may sometimes require more or less tokens than *k*. TD-NHG model utilizes top-p sampling to prevent the model from falling into sample from tail distribution. TD-NHG model should ensure that vocabulary probability distribution of the token after top-k sampling is greater than or equal to the baseline set by top-p sampling. Top-p sampling sets *p'* as a pre-defined constant $p^{\prime} \in (0,1)$, and *top-p* is set as 0.3 in this paper. Detailed comparison of ablation experiments is shown in Figs. 4 and 5 of “Results” chapter.

To improve the quality of generating news headlines and control the problem of generating duplicate words, the TD-NHG model uses the punishment mechanism. If word *v*_*i*_ has been selected to learn above, the probability of *v*_*i*_ being selected again will be reduced. The vocabulary was traversed to punish the probability of words appearing in the vocabulary sequence.

13 $$P(v_{i} ) = \sum\limits_{i = 1}^{m} {P(\left\{ { \, v_{1} ,v_{2} ,\ldots,v_{i} ,\ldots,v_{m} } \right\})} ,$$

14 $$P(v_{i} )^{\prime} = P(v_{i} )/repetition{\text{-}}penalty.$$

In this paper, penalty factor *repetition-penalty*=*1.2*, the use of Punishment Mechanism greatly improves the probability of the occurrence of secondary important words, reducing the probability of generating repeated words in news headlines, thus improving the accuracy of generating news headlines.

## Datasets and implementation details

### Datasets

This paper mainly solves the NHG problem, the TD-NHG model conducts comparative experiments on two news data sets to evaluate the model we designed. The first dataset is the LCSTS dataset^17^, which is created based on news summaries released by news media on microblogs. The total number of LCSTS data is 2,400,591. In this paper, the LCSTS dataset is integrated to remove duplicate data, text content words less than 100, and news headline words less than 2. After preprocessing, this paper obtains 1,331,209 experimental data, and uses the BertTokenizer to tokenize each utterance in the data. Randomly selected 3000 data as experimental test set, the remaining 1,328,209 as the training validation set. After preprocessing, the data information is as follows: the average number of words is 18, the standard deviation of words is 5, the maximum number of words is 30, and the minimum number is 4. The average number of words in the text is 104, the standard deviation is 10, the maximum number is 152, and the minimum number is 69.

The second dataset is the Chinese Short Text Summary (CSTS) dataset^27^, the original data is 670,000. CSTS dataset uses the same preprocessing method as LCSTS dataset. After preprocessing, the average number of words in news headlines is 20, the standard deviation of words is 6, the maximum number of words is 89, the minimum number is 4, an average number of words in news text is 125, standard deviation 31, maximum number 1749, minimum number 98. 446,877 news items were selected as the training and validation sets, and 3500 were selected as the test dataset. The basic properties of the data set are shown in Table 1.

**Table 1:** Dataset properties.

Dataset	# News articles	# News articles (after preprocessing)	Avg. # tokens per article	Avg. # tokens per headline
LCSTS	2,400,591	1,331,209	104	18
CSTS	670,000	450,377	125	20

### Implementation details

The model was trained in Nvidia 3060 (12G), as illustrated in Table 2. TD-NHG model uses 12 layers of improved transformer-decoder layers. We adopted the Adamw^28^ optimization algorithm, the Adamw optimizer's ambiguity factor (epsilon) was set to *1e−8*, the learning-rate was set to 1e−5, the warmup probability was 0.1, and every 4000 steps of training were tested. The random seed was set to 2020. For the LCSTS dataset and CSTS dataset, the length of the news input by the model was limited to 512, and the maximum length of the generated headline was 20. Mask multi-head self-attention has 12 heads, and a 12-layer improved decoding module was used. When calculating the loss, we defined the CrossEntropyLoss loss function, ignored the index of the calculated loss and the 0 loss in shift-labels, and only calculated the loss value for the news headline section. During the training stage, batch-size was set to 16 with 10 epochs. In the decoding process, top-k and top-k are used as the decoding search method. TD-NHG model set *top-k* = 6, *top-k* = 0.3, the repeated penalty rate was 1.2 and the vocabulary was 13,317.

**Table 2:** Experimental environment.

Experimental environment	Experimental configuration
Operating system	Ubuntu18.04
Programming language	Python3.8
Deep Learning Framework	Pytorch1.8.1
Display card model	Nvidia 3060 and Nvidia 3080ti (12G)

### Baseline model

The baseline models used in this paper include:

- RNN: RNN^17^ is based on the seq2seq model and does not use technical methods such as attention mechanisms,
- TextRanK: TextRanK^20^ is a retrieval-based text generation method that focuses on the proportion of sentences between each news positive and reorders them to generate headlines,
- ABS: ABS^13^ used an attention mechanism model based on the traditional seq-2-seq model, which is a common baseline model for generative text generation,
- CopyNet: CopyNet^8^ integrated the replication mechanism into the seq-2-seq model,
- HG-News: HG-News^29^ also used the transformer-decoder layer structure, enriched the model input module, added the personalized input module, and fused the pointer network in the model coding module,
- LSTM + Point: LSTM + Point^14^ Combined with pointer generator network in LSTM model. When generating a summary, the model can extract words from the original text to make the summary more accurate,
- LSTM + Point + Coverage: The coverage mechanism and pointer network are added based on the sep-2-sep generator model. The model effectively solves the out-of-vocabulary (OOV)^14^ problem and the problem of generating duplicate words by the generator. It is a conventional baseline model for generative headline generators.

### Evaluation

Recall-oriented under study for gisting evaluation (Rouge)^30^ is a set of important indicators used to evaluate machine translation and automatic text summarization. This paper compares the news headlines generated by news headline generation with the news headlines written by human beings from the original text and evaluates the news headlines based on the co-occurrence information of n-grams in the news text. Rouge is an evaluation index for the recall rate of n-gram words. The quality of news headlines is evaluated by counting the number of overlapping basic units (n-gram grammar, word sequence, and word pair) between the two. We take advantage of Rouge-N (including Rouge-1 and Rouge-2) and Rouge-L to score our model and compare these scores with other models proposed in the past. Rouge-N is defined as follows,

15 $$Rouge{\text{-}}N = \frac{{\mathop \sum \nolimits_{{S \in \left\{ {{\text{Re}} f} \right\}}} \mathop \sum \nolimits_{{gram_{n} \in S}} Count_{match} \left( {gram_{n} } \right)}}{{\mathop \sum \nolimits_{{S \in \left\{ {{\text{Re}} f} \right\}}} \mathop \sum \nolimits_{{gram_{n} \in S}} Count\left( {gram_{n} } \right)}}.$$

where n represents the length of the n-gram, $\mathop \sum \nolimits_{{S \in \left\{ {{\text{Re}} f} \right\}}} \mathop \sum \nolimits_{{gram_{n} \in S}} Count_{match} \left( {gram_{n} } \right)$ represents the sum of the number of n-grams in candidate news headlines and reference news headlines, and $\mathop \sum \nolimits_{{S \in \left\{ {Ref} \right\}}} \mathop \sum \nolimits_{{gram_{n} \in S}} Count\left( {gram_{n} } \right)$ represents the sum of the number of n-grams in reference news headlines. Rouge-N is a calculation method based on the recall rate, so the denominator of its calculation is the number of all n-grams in the reference headline set. The calculation formulas of Rouge-1 and Rouge-2 are introduced below,

16 $$Rouge{\text{-}}1 = \frac{{\mathop \sum \nolimits_{{S \in \left\{ {{\text{Re}} f} \right\}}} \mathop \sum \nolimits_{{\left( {1 {\text{-}} gram} \right) \in S}} Count_{match} \left( {1 {\text{-}} gram} \right)}}{{\mathop \sum \nolimits_{{S \in \left\{ {{\text{Re}} f} \right\}}} \mathop \sum \nolimits_{{\left( {1 {\text{-}} gram} \right) \in S}} Count\left( {1 {\text{-}} gram} \right)}},$$

17 $$Rouge{\text{-}}2 = \frac{{\mathop \sum \nolimits_{{S \in \left\{ {{\text{Re}} f} \right\}}} \mathop \sum \nolimits_{{\left( {2 {\text{-}} gram} \right) \in S}} Count_{match} \left( {2 {\text{-}} gram} \right)}}{{\mathop \sum \nolimits_{{S \in \left\{ {{\text{Re}} f} \right\}}} \mathop \sum \nolimits_{{\left( {2 {\text{-}} gram} \right) \in S}} Count\left( {2 {\text{-}} gram} \right)}}.$$

This paper adopted the Rouge-L index to calculate the longest common subsequence (LCS) between the two test units of the generated news headlines and the reference news headlines of the designed model. The Rouge-L formula is as follows,

18 $$ROUGE{\text{-}}L = \frac{{\left( {1 + \beta^{2} } \right)R_{lcs} P_{lcs} }}{{R_{lcs} + \beta^{2} P_{lcs} }},$$

19 $$R_{lcs} = \frac{{LCS\left( {X,Y} \right)}}{m},$$

20 $$P_{lcs} = \frac{{LCS\left( {X,Y} \right)}}{n},$$

Among them, *X* stands for model generating news headlines, and *Y* represents the original reference news headlines. $LCS\left( {X,Y} \right)$ denotes the longest common subsequence length of the generated summary and the reference summary, m denotes the reference news length of the original text, *n* denotes the length of the model-generated summary, and *β* is the weight coefficient. $R_{lcs}$ and $P_{lcs}$ represent the recall rate and accuracy, respectively.

## Results

The experiment in this paper was carried out on the LCSTS dataset and Chinese Short Text Summary Dataset (CSTS). The LCSTS test set consists of 3000 news bodies and 3000 news headlines, and the CSTS dataset is composed of 3500 news bodies and 3500 news headlines. To make the experimental results more convincing, we averaged the experimental results and took the average of 10 experimental results as the final experimental data. We performed many comparative experiments and ablation experiments to verify the effectiveness of the proposed TD-NHG model in news headline generation in the LCSTS dataset and CSTS dataset.

Tables 3 and 4 show that, regardless of the LCSTS dataset and CSTS dataset, the TD-NHG model proposed in this paper has a significant improvement compared with the baseline model introduced above. Analyzing the news headlines generated by the different models, for example, the “LSTM + Point + Coverage” model, which pays more attention to learning the text information in the original news body and was introduced into the newly generated news headlines through the pointer insertion form. The TD-NHG model learns the semantic information of the original text in the abstractive, focuses more on the readability and authenticity of the news headlines, and summarizes the news document. In terms of Rouge-1 and Rouge-2, it is similar to “LSTM + Point + Coverage” proposed by Hu et al.^17^, and slightly improved in Rouge-L, indicating that there is only still room for the improvement in readability of news headlines generated by TD-NHG model.

**Table 3:** Comparison of different models on LCSTS dataset.

Methods	Rouge-1			Rouge-2			Rouge-L		
	P	R	F	P	R	F	P	R	F
RNN	5.29	7.22	6.1	2.31	3.57	2.8	4.59	7.52	5.7
RNN-context	9.86	11.95	10.81	6.45	8.41	7.3	9.56	12.21	10.72
HG-news	–	–	22.79	–	–	7.7	–	–	21.36
ABS	–	–	28.15	–	–	11.07	–	–	25.35
LSTM + point	28.66	29.51	29.08	**13.81**	**15.74**	**14.71**	26.77	29.01	27.85
LSTM + point + coverage	**30.35**	**32.87**	**31.56**	11.64	13.88	12.66	27.05	29.09	28.03
TD-NHG (with top-k and top-p and punishment)	30.18	32.46	31.28	11.65	13.92	12.68	**27.32**	**29.38**	**28.31**

Significant values are given in bold.

**Table 4:** Comparison of different models on the CSTS dataset.

Methods	Rouge-1			Rouge-2			Rouge-L		
	P	R	F	P	R	F	P	R	F
RNN	18.72	20.48	19.56	7.93	9.59	8.68	16.23	18.62	17.34
ABS	–	–	30.14	–	–	14.07	–	–	27.35
TextRanK	31.22	33.51	32.32	14.58	15.51	15.03	25.66	26.81	26.22
CopyNet	34.11	34.74	34.28	20.17	22.62	21.32	30.17	32.21	31.15
LSTM + point	38.57	39.22	37.89	24.24	25.67	24.93	34.04	36.32	35.12
LSTM + point + coverage	38.72	**39.31**	**39.01**	**24.82**	**26.03**	**25.41**	36.79	38.04	37.40
TD-NHG (with greedy algorithm)	35.12	36.18	35.64	21.09	21.34	21.21	33.91	34.32	34.11
TD-NHG (with beam search = 4)	37.06	35.63	36.33	22.84	20.92	21.84	35.71	34.15	34.91
TD-NHG (with top-k and top-p, without punishment)	37.39	37.91	37.65	23.23	23.84	23.54	36.51	36.96	36.73
TD-NHG (with top-k = 6, top-p = 0.3 and punishment = 1.2)	**38.79**	38.68	38.73	24.79	25.15	24.97	**36.79**	**38.18**	**37.47**

Significant values are given in bold.

We performed ablation experiments on the LCSTS dataset for *top-k* and *top-p* on the TD-NHG model, as shown in Figs. 4 and 5. The influence of decoding selection strategy with different *top-k* and *top-p* on news headline generation is compared and analyzed under the same Punishment. Analyze whether the selection of *top-k* and *top-p* will affect the attention of the headline generator to words, thereby affecting the accuracy and readability of the news headline. We found that when $top{\text{-}}k = 6$ and cumulative probability $top{\text{-}}p = 0.3$, the Rouge index has the best effect, and headline generation has the best ability to describe the semantic features of news text. On the LCSTS dataset, the Rouge-1, Rouge-2, and Rouge-L indexes reached 31.28, 12.68, and 28.31, respectively. The Rouge index under different hyperparameters was significantly improved.

The third line of Table 4 proves that the decoding selection strategy of the punishment mechanism (repetition-penalty), *top-k* and *top-p* is effective in the task of headline generation, but there is no clear stipulation on how to select *top-k* , *top-p* and repetition-penalty. Therefore, this paper conducts ablation experiments on the top index. Ablation experiments show that in the news headline generation task, the same decoding selection strategy uses different restrictive criteria to have a significant impact on headline generation. When the *top-p* index is constant (e.g. $top{\text{-}}p = 0.3$), the *top-k* index affects the Rouge index used in this paper, as shown in Fig. 4. A comparison shows that when $top{\text{-}}k = 6$, the Rouge indexes reach the highest value, where Rouge-1 = 31.28, Rouge-2 = 12.68 and Rouge-L = 28.31. When $top{\text{-}}k$ index is constant (e.g. $top{\text{-}}k = 6$), the change of the *top-p* index is shown in Fig. 5. When $top{\text{-}}p = 0.3$, Rouge reaches the maximum.

**Figure 6:**
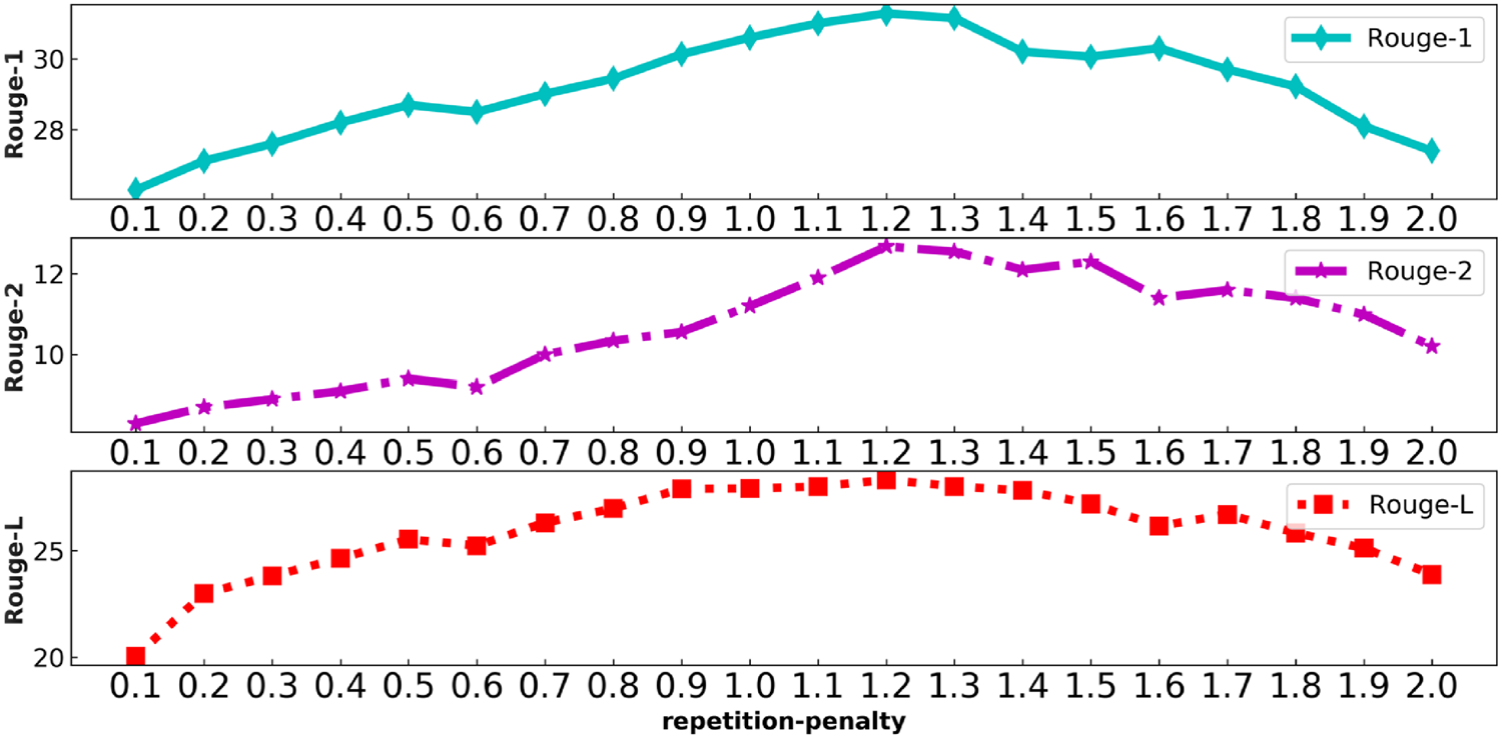
The influence curve of repetition-penalty on Rouge ($top{\text{-}}k = 6 \; and \;top{\text{-}}p = 0.3$).

We regulated the setting of Punishment (*repetition-penalty*) of the TD-NHG model by ablation experiments, as shown in Fig. 6. In the case of constant *top-k* and *top-p*, the TD-NHG model performed ablation experiments in the LCSTS dataset by continuously adjusting the repetition-penalty index. The news headline generator is prone to OOV problems when selecting the probability distribution of the vocabulary. TD-NHG model uses Punishment (repetition-penalty) to punish those words that have been selected many times. Figure 6 shows that the Rouge-1, Rouge-2 and Rouge-L indexes reach top at *repetition-penalty* = *1.2*.

## Discussion and case analysis

Tables 3 and 4 prove that our TD-NHG model has been further improved in the Rouge index, surpassing most existing headline generation models. When *batch-size*=*16* is set to train the LCSTS dataset, only approximately 75H hours are needed for 10 periods. Compared with all baseline models, the time cost is greatly reduced, and computing power is effectively saved. As shown in Figs. 4, 5, and 6, we utilized ablation experiments to verify how the decoding selection strategy in the TD-NHG model selected the hyperparameters accomplishments.

Table 5 shows some examples of the LCSTS dataset. By comparison, it is found that the TD-NHG model fully demonstrates the characteristics of abstractive headline generation. For example, the original news headline 'Worrying about Online Shopping “a double twelfth day” Fighting out Women's Intentional Remittance to Cheaters', the TD-NHG model designed in this paper generates the headline in the form of the subject-predicate object, “Women's Online Shopping Cheated Remittance ‘security account’ ”, which ensures the connectivity and readability of the news headline, and does not lack the necessary feature semantic information in the headline. However, because the abstractive model translates some learned semantic information, for the entity words that are not in the vocabulary, such as the original news headline in Table 7, “follow Uncle Xi to travel greatly”, where Uncle Xi is greatly a specific entity noun, there is no clear definition in the vocabulary. The poor learning effect of the model leads to a rough score lower than the average value, which greatly affects the overall rough score. For Tables 6, 7, a news headline containing specific entity nouns (including names, place names, etc.), which does not exist in the model vocabulary, we consider adding personalized input to the model input module or introducing external knowledge into the decoding to tackle this problem in future work.

**Table 5:** News headline generation examples on the TD-NHG model.

Original news	“Police comrade, this silly girl must send money to the liar, you hurry to help me persuade her… …” On the 12th, a middle-aged woman led a young woman into the police station. The young woman was in Taobao “a double twelfth day” **online shopping** received Telecomm uni-cations fraud text messages, for fear of 'accomplishments' to pay off, insisted on sending money to a **“security account”**
Original headline	Worrying about “a double twelfth day” online shopping women insist on remittance to cheaters
TD-NHG: top-k = 6, top-p = 0.3 and punishment = 1.2	**Women's Online Shopping Cheating, Adhere to the remittance to the “security account”**

Significant values are given in bold.

**Table 6:** News headline generation ep.1 using different TD-NHG parameters.

Original news	Having a home of its own has always been an important part of the **American dream**. Nevertheless, the proportion of adults with housing has been declining. According to commercial insiders, US **housing** ownership has been falling over the past few years, while rents have been rising and vacancy rates have been falling, suggesting a shift from **buying** to rent
Original headline	Can't afford a house The American dream is dying
TD-NHG: top-k = 6,top-p = 0.3 and punishment = 1.2	The truth of the **American Dream**: **Buying a house is transformed into renting a house**
TD-NHG: top-k = 2, top-p = 0.3 and punishment = 1.2	The Truth of **American Dream**: The Change of **Housing**
TD-NHG: top-k = 6, top-p = 0.95 and punishment = 1.2	The “**American Dream**” of the middle class **buying** houses

Significant values are given in bold.

**Table 7:** News headline generation ep. 2 using different TD-NHG parameters.

Original news	**Sri Lanka** 7 days free travel, 4557 yuan (Shanghai departure)! Known as "Tears on the Indian Ocean," **Sri Lanka** has beauteous beaches, a thousand-year-old ancient city, a Dutch castle, and rich tropical flora and fauna. Here, drinking a glass of authentic black tea, taking a water train ride, watching stilt fishermen fishing leisurely, going to the Lion Rock, and exploring the lost palace
Original headline	Follow Uncle Xi to travel greatly
TD-NHG: top-k = 6, top-p = 0.3 and repetition-penalty = 1.2	**Sri Lanka-**free travel
TD-NHG: top-k = 2, top-p = 0.3 and repetition-penalty = 1.2	Paradise crossing the train
TD-NHG: top-k = 6, top-p = 0.95 and repetition-penalty = 1.2	Traveling through **Sri Lanka**

Significant values are given in bold.

## Conclusion

In this paper, we proposed a novel news headline generation TD-NHG, which abandons the encoder-decoder structure used by the transformer and only utilizes 12 layers of improved transformer-decoder layers as the coding module. To learn the speech information and semantic features in the input token more accurately and quickly, the TD-NHG model adopted a masked multi-head self-attention mechanism and layer normalization layer in the coding module to obtain the attention distribution of the input token more accurately. In the TD-NHG model, we introduce different decoding selection strategies, including top-k, top-p, and the punishment mechanism (repetition-penalty), to select the words of news headlines. Experiments on the LCSTS dataset and CSTS dataset show that the TD-NHG proposed in this paper has achieved comparable results. In future work, we will consider solving the problem of out-of-vocabulary in news headline generation and the issue of inaccurate wording in the model when generating news headlines, thereby improving the semantic feature description ability and abstraction ability of the news headline generation.

## Data Availability

The datasets generated during or analysed during the current study are available from the corresponding author on reasonable request.
